# The gamma-glutamyl transpeptidase to platelet ratio for non-invasive assessment of liver fibrosis in patients with chronic hepatitis B and non-alcoholic fatty liver disease

**DOI:** 10.18632/oncotarget.16162

**Published:** 2017-03-13

**Authors:** Qiang Li, Chuan Lu, Weixia Li, Yuxian Huang, Liang Chen

**Affiliations:** ^1^ Department of Hepatitis, Shanghai Public Health Clinical Center, Fudan University, Shanghai 201508, China; ^2^ Department of Infectious Diseases, Huashan Hospital, Fudan University, Shanghai 200040, China

**Keywords:** chronic hepatitis B, non-alcoholic fatty liver disease, gamma-glutamyl transpeptidase-to-platelet ratio, liver fibrosis, non-invasive marker

## Abstract

**Background/Aim:**

The gamma-glutamyl transpeptidase-to-platelet ratio (GPR) is a novel serum model, which was reported more accurate than aspartate transaminase-to-platelet ratio index (APRI) and fibrosis index based on four factors (FIB-4) for diagnosing significant fibrosis and cirrhosis in HBV mono-infection in West Africa. We aimed to evaluate the diagnostic performance of GPR for liver fibrosis in patients with chronic hepatitis B (CHB) and non-alcoholic fatty liver disease (NAFLD).

**Results:**

Of 131 patients, 41 (31.3%), 20 (15.3%), and 10 (7.6%) were classified as having significant fibrosis, severe fibrosis and cirrhosis, respectively. To predict significant fibrosis, the AUROC of GPR was higher than that of APRI (0.86 vs 0.75, *p* = 0.001) and FIB-4 (0.86 vs 0.66, *p* < 0.001). To predict severe fibrosis, the AUROC of GPR was also higher than that of APRI (0.89 vs 0.77, *p* = 0.002) and FIB-4 (0.89 vs 0.72, p = 0.001). To predict cirrhosis, no difference was found between the AUROC of GPR and that of APRI (0.92 vs 0.86, *p* = 0.104).

**Materials and Methods:**

131 patients with CHB-NAFLD were included, and the diagnostic performances of GPR, APRI and FIB-4 were compared by receiver operating characteristic (ROC) curves and the area under ROC curves (AUROCs).

**Conclusions:**

The GPR could be used as a non-invasive marker to predict liver fibrosis and cirrhosis in CHB-NAFLD individuals.

## INTRODUCTION

Chronic hepatitis B (CHB), a disease caused by hepatitis B virus (HBV), is a leading cause for cirrhosis and hepatocellular carcinoma (HCC) [1]. Non-alcoholic fatty liver disease (NAFLD) covers a spectrum ranging from simple steatosis to steatohepatitis, and cirrhosis [2]. The prevalence of CHB is approximately 0.5% in the United States, 7% in China, and 10% in African countries [1]. The prevalence of NAFLD varies from 20% to 51%, depending on the study population [3]. The increasing prevalence of NAFLD has resulted in the increased coexistence of CHB and NAFLD. In developed countries, NAFLD was observed in 20% of CHB patients [4, 5].

Lemoine et al propose a novel fibrosis model—the gamma-glutamyl transpeptidase (GGT) to platelet ratio (GPR)—as a routinely available test that could identify patients with significant fibrosis or cirrhosis with higher diagnostic performance than aspartate transaminase (AST)-to-platelet ratio index (APRI) and fibrosis index based on four factors (FIB-4) in HBV mono-infection patients in West Africa [6]. Schiavon and colleagues subsequently reported that the GPR showed an acceptable diagnostic performance for the detection of liver fibrosis in Brazilian patients with CHB, but it does not add any advantage over APRI and FIB-4 [7]. In the subsequent study, Lemoine confirmed that the GPR predicts significant fibrosis and cirrhosis with higher diagnostic performance than APRI and FIB-4, in a large cohort of 721 HBV mono-infected Gambian patients using Fibroscan measures as a reference [8]. The heterogeneous populations may explain partly the discrepancies. Most of patients in the study by Schiavon et al are HBeAg seropositive (53%) and high HBV DNA levels (median, 5.0 log10 copies/ml) [7]; however, most of patients in the study by Lemoine et al. are HBeAg seronegative (96%) and low HBV DNA levels (median, 2.6 log10 copies/ml) [6]. The heterogeneous references for liver fibrosis may be another reason for the discrepancies. In the study by Schiavon et al, the evaluation of liver fibrosis was based on liver biopsy [7]; however, the 721 HBV mono-infected patients in the study by Lemoine et al used Fibroscan measures as a reference [8].

The above-mentioned studies excluded conditions that might predispose to altered GGT or platelet counts, including excessive alcohol consumption, accompanied by NAFLD, co-infection with HCV, HDV or HIV, and so on [6]. Consequently, high diagnostic accuracy of the GPR may not be applicative in patients with such conditions. Some researchers have done studies to validate the diagnostic accuracy of the GPR in CHB patients with such conditions. For example, Boyd et al reported that in a French HBV/HIV co-infected cohort, the GPR showed reasonable performance for identifying significant liver fibrosis [9]. Stockdale and colleagues subsequently reported that the GPR shows poor correlation with FibroScan measurements of liver fibrosis in HBV/HIV co-infected patients in West Africa [10].

At present, few studies have evaluated the performance of GPR for the diagnosis of liver fibrosis and cirrhosis in patients with CHB and NAFLD (CHB-NAFLD). To fill this research gap, we evaluated the diagnostic performance of GPR for significant fibrosis, severe fibrosis, and cirrhosis in one hundred and thirty-one CHB-NAFLD patients, and compared with APRI and FIB-4 scores.

## RESULTS

### Baseline characteristics of the patients

The baseline characteristics of enrolled patients were shown in Table 1. The majority of enrolled patients were male (72.5%), HBeAg positive (66.4%), and middle-aged (39 ± 10 years). The median HBV DNA, ALT, AST, and GGT were 5.6 log10 copies/ml (IQR = 3.5–7.5), 47 IU/L (IQR = 29–7 0), 30 IU/L (IQR = 24–37), and 45 IU/L (IQR = 16–5 7), respectively; and the mean platelet count was 182×10^9^/L. The Median GPR, APRI, and FIB-4 scores were 0.40 (IQR = 0.17–0.77), 0.44 (IQR = 0.32–0.69), and 0.98 (IQR = 0.68–1.43).

**Table 1:** Baseline characteristics of the study population

	Total (*n* = 131)
Male (*n*, %)	95 (72.5%)
Age (year)	39 ± 10
HBeAg positive, *n* (%)	87 (66.4%)
HBV DNA (log10 copies/ml)	5.6 (3.5–7.5)
ALT (IU/L)	47 (29–70)
AST (IU/L)	30 (24–37)
GGT (IU/L)	45 (16–57)
Platelet count (10^9^/L)	182 ± 61
GPR	0.40 (0.17–0.77)
APRI	0.44 (0.32–0.69)
FIB-4	0.98 (0.68–1.43)
Median BMI (kg/m2)	26 (23–28)
METAVIR Inflammation stage (A0/A1/A2/A3)	11 (8.4%)/47 (35.9%)/58 (44.3%)/15 (11.5%)
METAVIR Fibrosis stage (F0/F1/F2/F3/F4)	13 (9.9%)/77 (58.8%)/21 (16.0%)/10 (7.6%)/10 (7.6%)
Hepatic steatosis stage (G1/G2/G3/G4)	65 (49.6%)/41 (31.3%)/15 (11.5%)/10 (7.6%)

HBeAg, hepatitis B e antigen; ALT, alanine transaminase; AST, aspartate transaminase; GGT, gamma-glutamyl transpeptidase; GPR, GGT to platelet ratio index; APRI, AST to platelet ratio index; FIB-4, fibrosis index based on the 4 factors; BMI, body mass index.

The METAVIR inflammation stage was distributed as follows: A0 = 11 (8.4%); A1 = 47 (35.9%); A2 = 58 (44.3%); and A3 = 15 (11.5%). The METAVIR fibrosis stage was distributed as follows: F0 = 13 (9.9%); F1 = 77 (58.8%); F2 = 21 (16.0%); F3 = 10 (7.6%); and F4 = 10 (7.6%). The hepatic steatosis stage was distributed as follows: G1 = 65 (49.6%); G2 = 41 (31.3%); G3 = 15 (11.5%); and G4 = 10 (7.6%). Of 131 enrolled patients, 41 (31.3%), 20 (15.3%), and 10 (7.6%) were classified as having significant fibrosis, severe fibrosis, and cirrhosis, respectively.

### Correlations between noninvasive markers and METAVIR fibrosis stages

**Figure 1:**
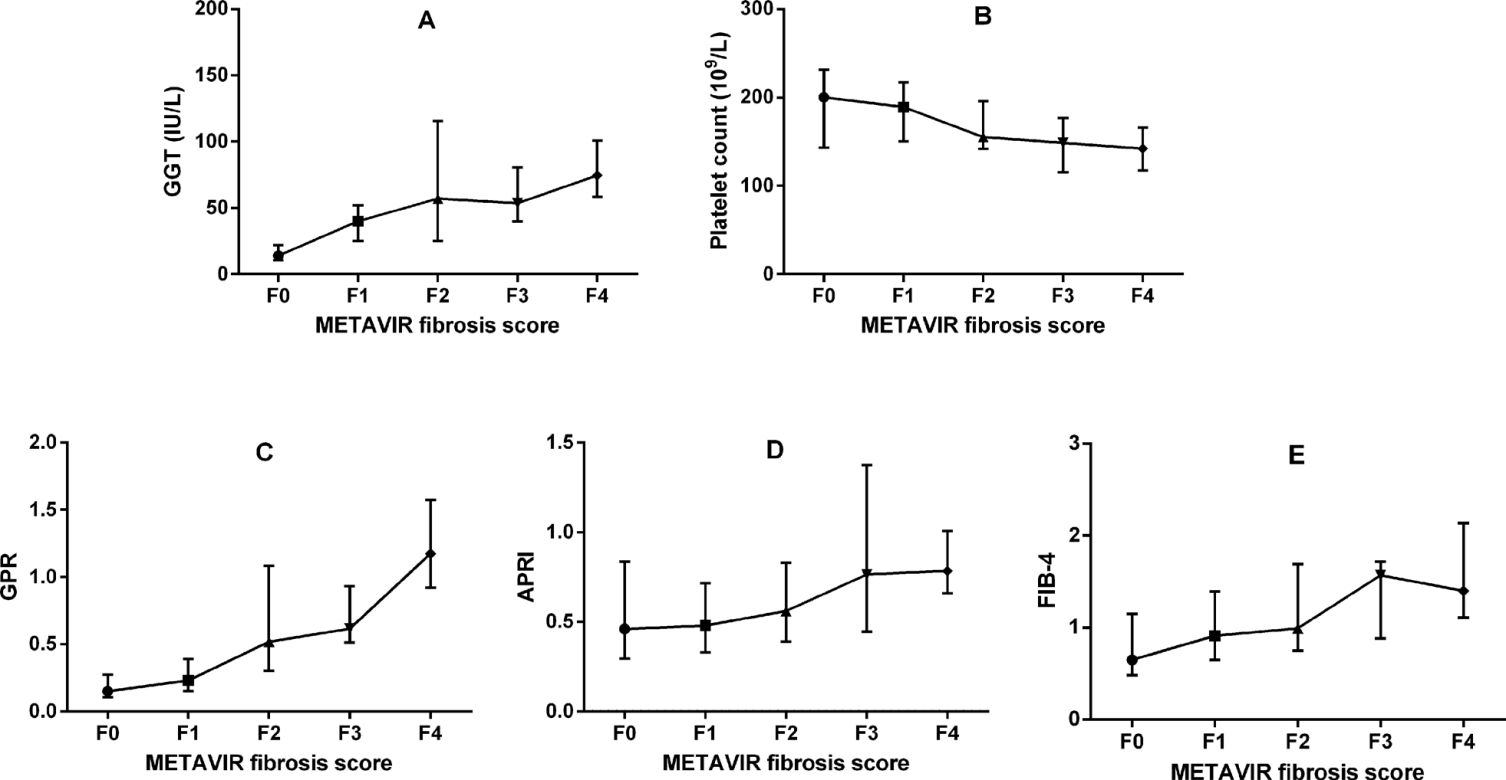
Association between METAVIR fibrosis stages and noninvasive markers GGT, gamma-glutamyl transpeptidase; GPR, GGT to platelet ratio index; APRI, aspartate transaminase to platelet ratio index; FIB-4, fibrosis index based on the 4 factors.

The correlations of noninvasive markers with METAVIR fibrosis stages were analysed using the Spearman test (Table 2). The GGT levels had a positive correlation with METAVIR fibrosis stages (correlation coefficient r = 0.57, *p* < 0.001), platelet count was negatively correlated (r = −0.32, *p* < 0.001). The METAVIR fibrosis stages were positive correlated with GPR (r = 0.60, *p* < 0.001), APRI (r = 0.39, *p* < 0.001), and FIB-4 (r = 0.25, *p* = 0.004). The association between METAVIR fibrosis stages and noninvasive makers was presented in Figure 1.

**Table 2:** Correlations between noninvasive markers and liver fibrosis stages

Variables	Spearman's r	*P* value
GGT (IU/L)	0.57	**< 0.001**
Platelet count (10^9^/L)	−0.32	**< 0.001**
GPR	0.60	**< 0.001**
APRI	0.39	**< 0.001**
FIB-4	0.25	**0.004**

GGT, gamma-glutamyl transpeptidase; GPR, GGT to platelet ratio index; APRI, aspartate transaminase to platelet ratio index; FIB-4, fibrosis index based on 4 factors; Spearman's r, correlation coefficient.

### Correlations between noninvasive markers and liver steatosis levels

The correlations of noninvasive markers with liver steatosis levels were presented in Table 3. The liver steatosis levels had no correlation with GGT (r = 0.03, *p* = 0.701), platelet count (r = −0.13, *p* = 0.135), GPR (r = 0.08, *p* = 0.389), APRI (r = 0.13, *p* = 0.142), and FIB-4 (r = 0.15, *p* = 0.098).

**Table 3:** Correlations between noninvasive markers and liver steatosis levels

Variables	Spearman's r	*P* value
GGT (IU/L)	0.03	0.701
Platelet count (10^9^/L)	−0.13	0.135
GPR	0.08	0.389
APRI	0.13	0.142
FIB-4	0.15	0.098

GGT, gamma-glutamyl transpeptidase; GPR, GGT to platelet ratio index; APRI, aspartate transaminase to platelet ratio index; FIB-4, fibrosis index based on 4 factors; Spearman's r, correlation coefficient.

### Diagnostic performances of noninvasive models for liver fibrosis and cirrhosis

**Figure 2:**
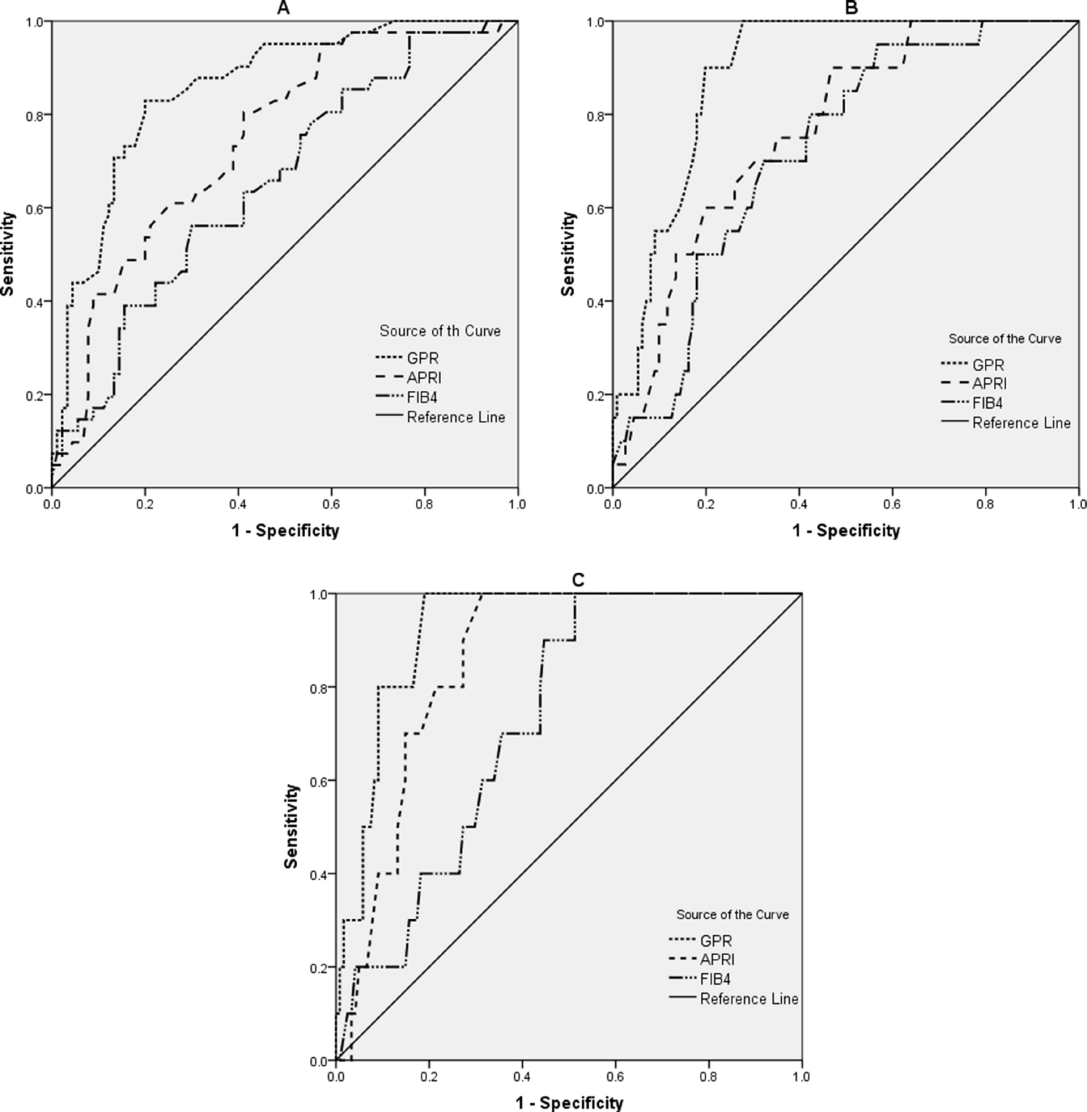
ROC curves for significant fibrosis (**A**), severe fibrosis (**B**), and cirrhosis (**C**). GPR, gamma-glutamyl transpeptidase to platelet ratio index; APRI, aspartate transaminase to platelet ratio index; FIB-4, fibrosis index based on the 4 factors.

The ROC curves of GPR, APRI, and FIB-4 for significant fibrosis (A), severe fibrosis (B), and cirrhosis (C), were shown in Figure 2. After estimating the performance to predict significant fibrosis, the AUROC of GPR was significantly higher than that of APRI (0.86 *vs* 0.75, *p* = 0.001) and FIB-4 (0.86 *vs* 0.66, *p* < 0.001) (Table 4). To predict severe fibrosis, the AUROC of GPR was also higher than that of APRI (0.89 *vs* 0.77, *p* = 0.002) and FIB-4 (0.89 *vs* 0.72, *p* = 0.001) (Table 4). For the diagnosis of cirrhosis, the AUROC of GPR was significantly higher than FIB-4 (0.92 *vs* 0.73, *p* < 0.001). However, no statistical difference was found between GPR and APRI (0.92 *vs* 0.86, *p* = 0.104) in predicting cirrhosis (Table 4).

**Table 4:** Diagnostic performances of noninvasive models for liver fibrosis and cirrhosis

	Significant fibrosis		Severe fibrosis		Cirrhosis	
	AUROC	(95% CI)	AUROC	(95% CI)	AUROC	(95% CI)
GPR	0.86	(0.79–0.91)	0. 89	(0.82–0.94)	0.92	(0.87–0.96)
APRI	0.75	(0.66–0.82)	0.77	(0.68–0.84)	0.86	(0.79–0.92)
FIB-4	0.66	(0.57–0.74)	0.72	(0.70–0.80)	0.73	(0.65–0.80)
Comparison of AUROC						
GPR and APRI	***p = 0.001***		***p = 0.002***		*p* = 0.104	
GPR and FIB-4	***p < 0.001***		***p = 0.001***		***p < 0.001***	
APRI and FIB-4	***p = 0.048***		*p* = 0.46		***p = 0.039***	

GPR, gamma-glutamyl transpeptidase to platelet ratio index; APRI, aspartate transaminase to platelet ratio index; FIB-4, fibrosis index based on the 4 factors; AUROC, the area under the receiver operating characteristic curve; CI, confidence interval.

### Diagnostic thresholds of noninvasive models for liver fibrosis and cirrhosis

Diagnostic thresholds of noninvasive models for liver fibrosis and cirrhosis were presented in Table 5. Maximizing Youden Index, the optimal cut-offs of GPR were 0.49, 0.62, and 0.74, for the diagnosis of significant fibrosis (the corresponding sensitivity, specificity, PPV, and NPV was 83%, 80%, 65%, and 91%, respectively), severe fibrosis (the corresponding sensitivity, specificity, PPV, and NPV was 90%, 75%, 39%, and 98%, respectively) and cirrhosis (the corresponding sensitivity, specificity, PPV, and NPV was 100%, 81%, 30%, and 100%, respectively), respectively. The optimal cut-offs of APRI were 0.41, 0.44, and 0.55, for the diagnosis of significant fibrosis (the corresponding sensitivity, specificity, PPV, and NPV was 80%, 59%, 47%, and 87%, respectively), severe fibrosis (the corresponding sensitivity, specificity, PPV, and NPV was 80%, 56%, 25%, and 94%, respectively) and cirrhosis (the corresponding sensitivity, specificity, PPV, and NPV was 100%, 69%, 21%, and 100%, respectively), respectively.

**Table 5:** Diagnostic thresholds of noninvasive models for liver fibrosis and cirrhosis

	Cut-offs	Youden Index	Sensitivity (%)	Specificity (%)	PPV (%)	NPV (%)	PLR	NLR
GPR								
≥ F2	0.49	0.63	83	80	65	91	4.15	0.21
≥ F3	0.62	0.65	90	75	39	98	3.57	0.13
= F4	0.74	0.81	100	81	30	100	5.26	0
APRI								
≥F2	0.41	0.39	80	59	47	87	1.96	0.33
≥ F3	0.44	0.36	80	56	25	94	1.81	0.36
= F4	0.55	0.69	100	69	21	100	3.18	0
FIB-4								
≥ F2	0.77	0.22	86	36	37	84	1.32	0.41
≥ F3	0.83	0.38	95	43	23	98	1.67	0.12
= F4	0.91	0.49	100	49	14	100	1.95	0

GPR, gamma-glutamyl transpeptidase to platelet ratio index; APRI, aspartate transaminase to platelet ratio index; FIB-4, fibrosis index based on the 4 factors; PPV, positive predictive value; NPV, negative predictive value; PLR, positive likelihood ratio; NLR, negative likelihood ratio. The GPR cut-offs obtained in HBV mono-infection patients in West Africa by Lemoine et al. were 0.32 for ≥ F2, 0.32 for ≥ F3, and 0.56 for = F4, respectively.

## DISCUSSION

HBV and NAFLD are highly co-endemic in China, and liver fibrosis is a common pathological process in CHB-NAFLD patients. Among CHB-NAFLD patients, those with significant fibrosis or cirrhosis are at increased risk for liver de-compensation, hepatocellular carcinoma (HCC), and death [1]. To reduce the disease burden, it is critical to identify patients with significant fibrosis or cirrhosis, and treat them timely. The early detection of significant fibrosis or cirrhosis is an essential step for CHB-NAFLD patients in deciding treatment commencement, course of treatment, and prognosis. Liver biopsy is the gold standard for assessment of liver fibrosis, but limited for its invasiveness, expensive procedure, and potentially complications.

In this study, we observed that the GPR had a higher performance compared to other commonly used models (APRI and FIB-4) for diagnosing significant fibrosis and severe fibrosis (all *p* < 0.05) in CHB-NAFLD patients. For the diagnosis of cirrhosis, no statistical difference was found between GPR and APRI (*p* = 0.104). This study indicated that the GPR, which shows application prospect in HBV mono-infection or HBV/HIV co-infection patients in West Africa, also could be used to identify significant fibrosis, severe fibrosis, and cirrhosis in CHB-NAFLD patients. In this study, the liver steatosis levels had no correlation with GGT, platelet count, and GPR, along with no correlation between steatosis and fibrosis, which might help explain why the GPR works in CHB-NAFLD patients.

The GPR cut-offs in this study (≥ F2, 0.49; ≥ F3, 0.62; = F4, 0.74) were higher than those obtained in HBV mono-infection patients in West Africa (≥ F2, 0.32; ≥ F3, 0.32; = F4, 0.56) [8]. As GGT levels were higher overall in CHB-NAFLD patients in this study, compared with HBV mono-infection patients in the study by Lemoine et al (45 *vs* 36 IU/L) [8], and the need for modified thresholds would likely stem from elevated GGT levels. Kumar et al found that the CHB-NAFLD patients tend to present with higher transaminase and GGT levels compared with HBV mono-infection [11]. In addition, obesity could increase GGT; and CHB-NAFLD patients in this study have higher BMI (26 *vs* 22 kg/m2) than HBV mono-infection patients in the study by Lemoine et al [8]. Secondly, the prevalence of fibrosis (31.3% for ≥ F2; 15.3% for ≥ F3; 7.6% for = F4) in this study was lower than what was obtained by Lemoine et al (39% for ≥ F2; 32% for ≥ F3; 15% for = F4) [8]. Difference between the GPR cut-offs may be related to differences in prevalence of liver fibrosis in the studied populations, known as the spectrum bias [12, 13].

GPR had good NPVs for excluding significant fibrosis (91%), severe fibrosis (98%), and cirrhosis (100%), respectively; but low PPVs for diagnosing significant fibrosis (65%), severe fibrosis (39%), and cirrhosis (30%), respectively. Likewise, in this study, APRI and FIB-4 also had low PPVs for diagnosing significant fibrosis (47% and 37%, respectively), severe fibrosis (25% and 23%, respectively), and cirrhosis (21% and 14%, respectively), respectively. In fact, the low PPVs were common problem with noninvasive fibrosis models. According to the recent WHO HBV guideline, the PPV was low (less than 50%) for all non-invasive tests for the diagnosis of liver fibrosis and cirrhosis, and FibroScan had a relatively higher PPV (42%) than APRI using either a high or low cut-off (26% and 22%) [14]. Although the PPVs of all noninvasive fibrosis models were low, GPR had a relatively higher PPVs compared with APRI and FIB-4, for the diagnosis of significant fibrosis (65%, 47%, and 37%, respectively), severe fibrosis (39%, 25%, and 23%, respectively), and cirrhosis (30%, 21%, and 14%, respectively), respectively.

It is important to note that the APRI threshold for the diagnosis of cirrhosis (> 2.0) recommended by the WHO HBV guideline was unsuitable in CHB-NAFLD population, such that none of the ten patients with cirrhosis were correctly identified. This implies that 100% of patients who had cirrhosis would be erroneously categorized as patients without cirrhosis by APRI > 2. In this study, the optimal cut-off of APRI is 0.55 to diagnose cirrhosis, and the corresponding sensitivity, specificity, PPV, and NPV were 100%, 69%, 21%, and 100%, respectively. Obviously, APRI > 0.55 is more appropriate for screening cirrhosis and selection of candidates for liver biopsy in CHB-NAFLD population. Compared with HBV monoinfection patients, the different magnitude of inflammation and related ALT levels observed in CHB-NAFLD patients, that might render the different APRI cut-offs. Two recent studies, showing different pathogenesis and different patterns of fibrosis according to different causes of chronic liver diseases, also justify the need for different cut-offs of systems for assessment of fibrosis from different causes [15, 16].

It is undeniable that this study has some limitations. First, there were few patients with F3 and F4 fibrosis (*n* = 20) and perhaps there was lack of power to determine a statistical difference between APRI and GPR AUROCs at the F4 level. Second, our study population, with higher prevalence of HBeAg-positivity and higher proportion of male, might not be fully representative of CHB-NAFLD patients. Third, we do not compare the performance of FibroScan to GPR because of the FibroScan measurements have not been promoted widely in China. Fourth, we do not compare the performance of FibroTest, Hepascore and FibroMeter to GPR because these models were protected by patents, and some laboratory tests which are necessary for the calculations of these models were not available in our hospital. Fifth, there is no external validation, and the predictive capacity of the GPR thresholds obtained in this study need to be further validated in other cohorts of similar study population.

Notwithstanding these limitations, we validate that the GPR could be used as a non-invasive marker to predict liver fibrosis and cirrhosis for CHB-NAFLD individuals, especially when compared with APRI and FIB-4. Certainly, we hope that further evaluations will be conducted in CHB-NAFLD patients more broadly.

## MATERIALS AND METHODS

### Study design and patients

**Figure 3:**
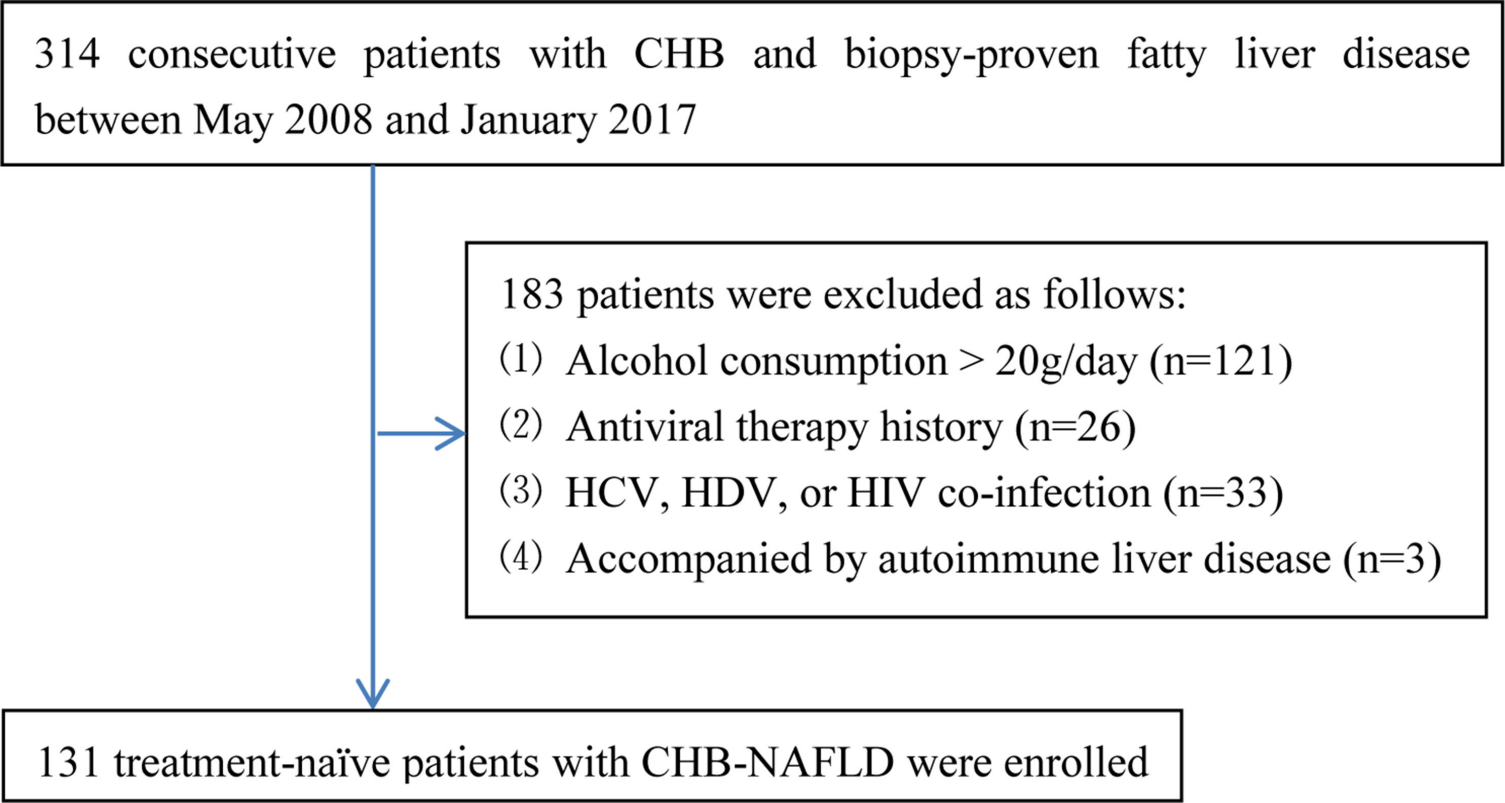
Flow diagram of the study population CHB, chronic hepatitis B; NAFLD, non-alcoholic fatty liver disease; HCV, hepatitis C virus; HDV, hepatitis D virus; HIV, human immunodeficiency virus.

Three hundred and fourteen consecutive patients with CHB and biopsy-proven fatty liver disease (defined as the presence of more than 5% steatosis of hepatocytes) who underwent liver biopsies and routine laboratory tests at Shanghai Public Health Clinical Center, Shanghai, China between May 2008 and January 2017 were retrospectively screened. CHB was defined as the persistent presence of hepatitis B surface antigen (HBsAg) for more than 6 months [17]. The fatty liver disease was considered to be non-alcoholic in origin if the clinical records indicated that the patient was felt to either be totally abstinent or to consume less than approximately 20 g of alcohol daily [18]. Patients with the following conditions were excluded: (1) alcohol consumption > 20 g/day (*n* = 121); (2) antiviral therapy history (*n* = 26); (3) co-infection with hepatitis C virus (HCV), hepatitis D virus (HDV), or human immunodeficiency virus (HIV) (*n* = 33); (4) accompanied by autoimmune liver disease (*n* = 3). Finally, 131 treatment-naïve patients with CHB-NAFLD were included. Figure 3 summarized the flow diagram of the study population.

All patients signed the informed consent before liver biopsy, and all clinical procedures were in accordance with the Helsinki declaration of 1975, as revised in 1983. The study protocol was permitted by the ethics committee of Shanghai Public Health Clinical Center.

### Liver histological score

Liver biopsy was performed using ultrasound localization. Liver samples were formalin-fixed and paraffin-embedded. A minimum of 15mm of liver tissue with at least 6 portal tracts is considered sufficient for liver histological scoring. Liver histology was interpreted by two liver pathologists. In case of discrepancies, slides were reviewed by a third highly experienced hepatopathologist.

The METAVIR scoring system was adopted as the histological standard of liver fibrosis, which was classified into five stages: F0, no fibrosis; F1, portal fibrosis without septa; F2, portal fibrosis with rare septa; F3, numerous septa without cirrhosis; and F4, cirrhosis [19]. Steatosis was defined as the presence of more than 5% steatosis of hepatocytes, and the degree of steatosis was semi-quantitatively graded from 0 to 4 as follows: grade 0: less than 5% steatosis; grade 1: 6% to 25%, grade 2: 26% to 50%, grade 3: 51% to 75%, and grade 4: more than 76% [20]. Significant fibrosis was defined as fibrosis stage ≥ F2, severe fibrosis was defined as fibrosis stage ≥ F3, and cirrhosis was defined as fibrosis stage = F4. Steatohepatitis was defined by the minimal criteria of hepatic steatosis and scattered, mainly lobular inflammation with or without Mallory bodies, cytologic ballooning, and perisinusoidal fibrosis [21].

### Routine laboratory tests

Fasting blood samples were obtained, and routine laboratory tests were performed the day before liver biopsy. The serological markers of HBV were detected with enzyme-linked immune-sorbent assay kits (ARCHITECT i2000 SR; Abbott, Wiesbaden, Germany). The serum biochemical parameters including alanine transaminase (ALT), AST, and GGT were measured by full automated biochemistry analyzer (7600 Series; Hitachi, Tokyo, Japan). Platelet count was detected with automated hematology analyzer (XT-2000i, Sysmex, Kobe, Japan). HBV DNA was quantified by real-time PCR (ABI 7500; Applied Biosystems, Foster City, USA) with the lowest detection limit at 500 copies/ml.

### Noninvasive models calculation

The formulas for GPR, APRI, and FIB-4 are as follows: (1) GPR = (GGT(IU/L)/ULN of GGT)/platelet count(10^9^/L)×100; (2) APRI = (AST(IU/L)/ULN of AST)/platelet count (10^9^/L)×100; (3) FIB-4 = (age(years)×AST (IU/L))/(platelet count (10^9^/L)×(ALT (IU/L))^1/2^).

Note: ULN of AST = 40 IU/L; ULN of GGT = 50 IU/L.

### Statistical analysis

Normality tests of baseline data were performed by Kolmogorov-Smirnov test. The baseline data of enrolled patients was presented as follows: normal distribution data as mean ± standard deviation, non-normal distribution continuous data as median (interquartile range (IQR)), and categorical variables as number (percentage). The diagnostic performances of GPR, APRI, and FIB-4 were evaluated and compared by the receiver operating characteristic (ROC) curves and the area under the ROC curves (AUROCs) [22]. The ROC curve analysis and *Z*-test was, respectively, used to compute and compare AUROC. The optimal cut-offs were obtained by maximizing Youden index (sensitivity+specificity-1). Diagnostic accuracy was evaluated by sensitivity, specificity, positive predictive value (PPV), negative predictive value (NPV), positive likelihood ratio (PLR), and negative likelihood ratio (NLR). All significance tests were two tailed, and *p* < 0.05 was considered statistically significant. All statistical analyses were carried out using the SPSS statistical software version 15.0 (SPSS Inc. Chicago, IL, USA) and MedCalc Statistical Software version 16.1 (MedCalc Software bvba, Ostend, Belgium).
